# 4,4′-Dichloro-2,2′-(piperazine-1,4-diyldimethyl­ene)diphenol

**DOI:** 10.1107/S1600536808035769

**Published:** 2008-11-13

**Authors:** Koji Kubono, Yuki Tsuno, Keita Tani, Kunihiko Yokoi

**Affiliations:** a Division of Natural Sciences, Osaka Kyoiku University, Kashiwara, Osaka 582-8582, Japan

## Abstract

In the titile compound, C_18_H_20_Cl_2_N_2_O_2_, the piperazine ring adopts a chair conformation. The mol­ecule has a non-crystallographic inversion centre in the middle of the piperazine ring at approximate position (3/4, 1/8, 3/8). There are intra­molecular O—H⋯N hydrogen bonds forming *S*(6) ring motifs. Inter­molecular C—H⋯O hydrogen bonds generate anti­parallel *C*(5) chain motifs propagating along the *b* axis, forming sheets parallel to the *bc* plane with a first-level graph-set *S*(6)*C*(5)*R*_6_^6^(26).

## Related literature

For graph-set notations for hydrogen bonds, see: Bernstein *et al.* (1995 ▶). For the synthesis of a ligand with two piperazine arms, see: Bharathi *et al.* (2006 ▶). For the use of piperazine derivatives as buffers, see: Good *et al.* (1966 ▶). For the monoclinic and ortho­rhom­bic polymorphs of a tetra­chloro-2,2′-(piperazine-1,4-diyldimethyl­ene)diphenol, see: Kubono & Yokoi (2007 ▶). For the structure of 1,4-bis­(2-hydr­oxy-5-methyl­benz­yl)piperazine, see: Kuppayee *et al.* (1999 ▶).
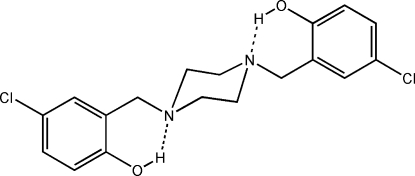


## Experimental

### 

#### Crystal data

C_18_H_20_Cl_2_N_2_O_2_
*M* *_r_* = 367.26
Orthorhombic, 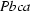

*a* = 14.055 (4) Å
*b* = 21.214 (11) Å
*c* = 11.873 (3) Å
*V* = 3540 (2) Å^3^
*Z* = 8
Mo *K*α radiation
μ = 0.38 mm^−1^
*T* = 298.1 K
0.18 × 0.13 × 0.13 mm

#### Data collection

Rigaku AFC-7R diffractometer
Absorption correction: none
5928 measured reflections
4066 independent reflections
2735 reflections with *F* ^2^ > 2σ(*F* ^2^)
*R*_int_ = 0.039
3 standard reflections every 150 reflections intensity decay: 0.7%

#### Refinement

*R*[*F* ^2^ > 2σ(*F* ^2^)] = 0.039
*wR*(*F* ^2^) = 0.105
*S* = 1.00
2739 reflections
237 parameters
All H-atom parameters refined
Δρ_max_ = 0.33 e Å^−3^
Δρ_min_ = −0.45 e Å^−3^

### 

Data collection: *WinAFC* (Rigaku/MSC, 2006 ▶); cell refinement: *WinAFC*; data reduction: *CrystalStructure* (Rigaku/MSC, 2006 ▶); program(s) used to solve structure: *SIR92* (Altomare *et al.*, 1993 ▶); program(s) used to refine structure: *CRYSTALS* (Betteridge *et al.*, 2003 ▶); molecular graphics: *PLATON* (Spek, 2003 ▶); software used to prepare material for publication: *CrystalStructure*.

## Supplementary Material

Crystal structure: contains datablocks global, I. DOI: 10.1107/S1600536808035769/si2120sup1.cif

Structure factors: contains datablocks I. DOI: 10.1107/S1600536808035769/si2120Isup2.hkl

Additional supplementary materials:  crystallographic information; 3D view; checkCIF report

## Figures and Tables

**Table 1:** Hydrogen-bond geometry (Å, °)

*D*—H⋯*A*	*D*—H	H⋯*A*	*D*⋯*A*	*D*—H⋯*A*
O1—H1⋯N1	0.85	1.88	2.649 (3)	150
O2—H20⋯N2	0.85	1.87	2.647 (3)	151
C7—H6⋯O2^i^	0.95	2.59	3.230 (3)	125
C12—H15⋯O1^ii^	0.95	2.56	3.300 (3)	134

Symmetry codes: (i) 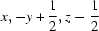; (ii) 
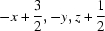
.

**Fig. 1.**
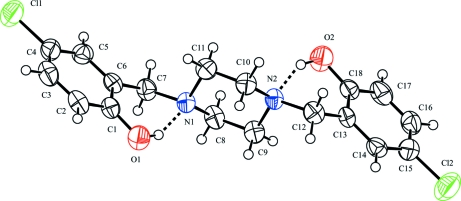
The molecular structure of (I) with the atom-labelling scheme and displacement ellipsoids are drawn at the 50% probability level. H atoms are represented by circles of arbitrary size.

**Fig. 2.**
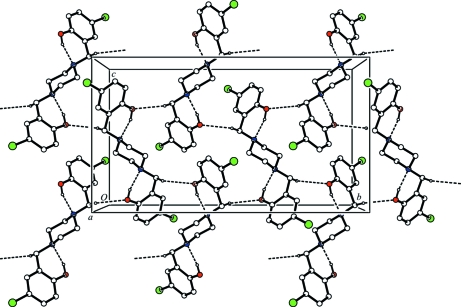
The molecular packing of (I), showing the formation of a sheet with a first level graph set S(6)C(5)R66(26). The hydrogen bonds are shown as dashed lines. The H atoms not involved in the hydrogen bonds have been omitted for clarity.

## supplementary crystallographic information

### Comment

Piprazine derivatives are widly utilized as buffers, *e.g.*, 4-(2-hydroxyethyl)-1-piperazineethanesulfonic acid (HEPES) (Good *et al.*, 1966), and can act as complexing reagents with metal ions (Bharathi *et al.*, 2006).

The molecular structure of the title compound (Fig. 1) (I), consists of two chlorophenol arms and a piperazine ring, which adopt a chair conformation. The molecule has a pseudo-inversion centre in the middle of the piperazine ring at position (3/4, 1/8, 3/8). It is interesting to note that in the polymorh structures of dichlorophenol derivatives (Kubono & Yokoi, 2007) the molecules occupy crystallographic inversion centres (*Z*' = 1/2). The bond lengths and angles in (I) are normal and comparable with those in the monoclinic and orthorhombic polymorph structures (Kubono & Yokoi, 2007) and in the *p*-cresol derivative (Kuppayee *et al.*, 1999). Intramolecular O—H···N hydrogen bonds in (I) have similar geometric parameters and higher level graph set notations as was observed in the polymorph structures. The torsion angles C1—C6—C7—N1 and N2—C12—C13—C18 are -34.8 (3) and 37.5 (3) °, respectively. The dihedral angles between the mean planes of two benzene rings are 4.68 (12) °.

In the crystal structure of (I), there are two intermolecular C—H···O hydrogen bonds (Table 1). Atom C7 in the molecule at (*x*, *y*, *z*) acts as hydrogen bond donor to atom O2 in the molecule at (*x*, 1/2 - *y*, *z* - 1/2), so forming a *C*(5) (Bernstein *et al.*, 1995) chain running parallel to the [010] direction and generated by the *c*-glide plane at *y* = 1/4. In addition, atom C12 in the molecule at (*x*, *y*, *z*) acts as hydrogen bond donor to atom O1 atom in the molecule at (3/2 - *x*, -*y*, 1/2 + *z*), so forming a *C*(5) chain running parallel to the [010] direction and generated by the 2_1_ screw axis along (3/4, 0, *z*). The molecules are linked by the combination of the two *S*(6) rings and the two antiparalle *C*(5) chains into a sheet parallel to *b*,*c*-plane with a first level graph set *S*(6)*C*(5)*R*_6_^6^(26) (Fig. 2).

### Experimental

A mixture of 4-chlorophenol (25.0 g, 194 mmol), piperazine (8.34 g, 97.2 mmol) and paraformaldehyde (5.82 g, 194 mmol) in methanol (80 ml) was refluxed for 6 h. The mixture was cooled to room temperature, then the solvent was evaporated under vacuum. The product was recrystallized from CHCl_3_—MeOH to give prismatic crystals of (I) [yeild 13.8 g (38.7%); m.p. 515.0–515.4 K]. Analysis calculated for C_18_H_20_Cl_4_N_2_O_2_: C 58.86, H 5.49, N 7.63%; found: C 58.50, H 5.44, N 7.55%. ^1^H-NMR(CDCl_3_, p.p.m., 400 MHz): 2.68 (*brs*, 8H, CH_2_), 3.69 (*s*, 4H, CH_2_), 6.75 (*d*, *J* = 2.4 Hz, 2H, ArH), 6.96 (*s*, 2H, ArH), 7.13 (*d*, *J* = 2.4 Hz, 2H, ArH), 10.6 (*brs*, 2H, OH).

### Refinement

The H atoms of the hydroxyl groups were found from a difference Fourier map. The other H atoms were placed at idealized positions with C—H = 0.95 Å. All the H atoms were refined as a riding model with *U*_iso_(H) = 1.2 *U*_eq_(C).

### Crystal data

**Table:**

C_18_H_20_Cl_2_N_2_O_2_	*F*_000_ = 1536.00
*M**_r_* = 367.26	*D*_x_ = 1.378 Mg m^−^^3^
Orthorhombic, *P**b**c**a*	Mo *K*α radiation λ = 0.71069 Å
Hall symbol: -P 2ac 2ab	Cell parameters from 18 reflections
*a* = 14.055 (4) Å	θ = 13.7–16.9º
*b* = 21.214 (11) Å	µ = 0.38 mm^−^^1^
*c* = 11.873 (3) Å	*T* = 298.1 K
*V* = 3540 (2) Å^3^	Prismatic, colorless
*Z* = 8	0.18 × 0.13 × 0.13 mm

### Data collection

**Table:**

Rigaku AFC-7R diffractometer	θ_max_ = 27.5º
ω scans	*h* = −10→18
Absorption correction: none	*k* = 0→27
5928 measured reflections	*l* = −8→15
4066 independent reflections	3 standard reflections
2735 reflections with *F*^2^ > 2σ(*F*^2^)	every 150 reflections
*R*_int_ = 0.039	intensity decay: 0.7%

### Refinement

**Table:**

Refinement on *F*^2^	All H-atom parameters refined
*R*[*F*^2^ > 2σ(*F*^2^)] = 0.039	*w* = 1/[0.0011*F*_o_^2^ + σ(*F*_o_^2^)]/(4*F*_o_^2^)
*wR*(*F*^2^) = 0.105	(Δ/σ)_max_ < 0.001
*S* = 1.00	Δρ_max_ = 0.33 e Å^−^^3^
2739 reflections	Δρ_min_ = −0.45 e Å^−^^3^
237 parameters	Extinction correction: none

### Special details

**Table:**

Geometry. The molecule adopts a non-crystallographic inversion centre in the middle of the piperazine ring at an approximate position (3/4, 1/8, 3/8).
Refinement. Refinement was performed using reflections with *F*^2^ > 2.0 σ(*F*^2^). The weighted *R*-factor (*wR*) and goodness of fit (*S*) are based on *F*^2^. *R*-factor (gt) are based on *F*. The threshold expression of *F*^2^ > 2.0 σ(*F*^2^) is used only for calculating *R*-factor (gt).

### Fractional atomic coordinates and isotropic or equivalent isotropic displacement parameters (Å^2^)

**Table:**

	*x*	*y*	*z*	*U*_iso_*/*U*_eq_	
Cl1	0.43588 (6)	0.27754 (4)	−0.07209 (8)	0.0807 (3)	
Cl2	1.07341 (7)	−0.01127 (5)	0.83011 (9)	0.0914 (4)	
O1	0.75085 (15)	0.11534 (9)	0.07111 (17)	0.0559 (7)	
O2	0.74244 (15)	0.13266 (9)	0.67705 (18)	0.0643 (8)	
N1	0.73683 (16)	0.16416 (11)	0.2758 (2)	0.0385 (7)	
N2	0.77032 (17)	0.08768 (10)	0.4717 (2)	0.0385 (8)	
C1	0.6764 (2)	0.15283 (15)	0.0418 (2)	0.0435 (10)	
C2	0.6298 (2)	0.14079 (15)	−0.0579 (2)	0.0506 (11)	
C3	0.5560 (2)	0.17790 (18)	−0.0936 (2)	0.0558 (12)	
C4	0.5283 (2)	0.22809 (16)	−0.0274 (3)	0.0526 (12)	
C5	0.5720 (2)	0.24037 (15)	0.0734 (2)	0.0477 (11)	
C6	0.6466 (2)	0.20327 (14)	0.1097 (2)	0.0398 (10)	
C7	0.6988 (2)	0.21952 (13)	0.2166 (2)	0.0459 (10)	
C8	0.8043 (2)	0.18345 (14)	0.3642 (2)	0.0483 (10)	
C9	0.8455 (2)	0.12615 (13)	0.4217 (2)	0.0465 (10)	
C10	0.7022 (2)	0.06864 (13)	0.3845 (2)	0.0451 (10)	
C11	0.6609 (2)	0.12632 (13)	0.3279 (2)	0.0466 (10)	
C12	0.8101 (2)	0.03329 (13)	0.5321 (2)	0.0476 (10)	
C13	0.8583 (2)	0.05200 (14)	0.6408 (2)	0.0381 (10)	
C14	0.9375 (2)	0.01930 (13)	0.6786 (2)	0.0451 (11)	
C15	0.9773 (2)	0.03340 (15)	0.7820 (3)	0.0505 (11)	
C16	0.9415 (2)	0.08039 (17)	0.8475 (2)	0.0544 (12)	
C17	0.8640 (2)	0.11345 (15)	0.8107 (3)	0.0563 (12)	
C18	0.8214 (2)	0.09977 (14)	0.7088 (2)	0.0432 (11)	
H1	0.7660	0.1247	0.1381	0.067*	
H2	0.6488	0.1057	−0.1022	0.061*	
H3	0.5249	0.1702	−0.1632	0.067*	
H4	0.5506	0.2748	0.1178	0.057*	
H5	0.6569	0.2415	0.2656	0.055*	
H6	0.7510	0.2460	0.1977	0.055*	
H7	0.8537	0.2074	0.3303	0.058*	
H8	0.7724	0.2085	0.4187	0.058*	
H9	0.8880	0.1390	0.4795	0.056*	
H10	0.8790	0.1019	0.3674	0.056*	
H11	0.7338	0.0442	0.3288	0.054*	
H12	0.6530	0.0443	0.4178	0.054*	
H13	0.6301	0.1509	0.3839	0.056*	
H14	0.6161	0.1145	0.2718	0.056*	
H15	0.7599	0.0047	0.5485	0.057*	
H16	0.8557	0.0132	0.4853	0.057*	
H17	0.9651	−0.0127	0.6331	0.054*	
H18	0.9701	0.0897	0.9181	0.065*	
H19	0.8387	0.1466	0.8555	0.068*	
H20	0.7346	0.1261	0.6069	0.077*	

### Atomic displacement parameters (Å^2^)

**Table:**

	*U*^11^	*U*^22^	*U*^33^	*U*^12^	*U*^13^	*U*^23^
Cl1	0.0581 (6)	0.0946 (8)	0.0893 (8)	0.0015 (5)	−0.0153 (6)	0.0291 (5)
Cl2	0.0782 (7)	0.1153 (8)	0.0806 (8)	0.0288 (6)	−0.0241 (6)	−0.0050 (6)
O1	0.0720 (15)	0.0498 (13)	0.0459 (15)	0.0121 (12)	−0.0003 (12)	−0.0089 (12)
O2	0.0803 (17)	0.0591 (14)	0.0534 (17)	0.0224 (13)	−0.0010 (13)	−0.0097 (12)
N1	0.0419 (15)	0.0361 (14)	0.0377 (17)	−0.0053 (13)	−0.0039 (13)	0.0012 (13)
N2	0.0441 (16)	0.0304 (14)	0.0410 (17)	−0.0081 (13)	−0.0031 (13)	0.0035 (13)
C1	0.049 (2)	0.041 (2)	0.040 (2)	−0.0044 (18)	0.0014 (18)	0.0048 (18)
C2	0.064 (2)	0.050 (2)	0.037 (2)	−0.014 (2)	0.005 (2)	0.0001 (19)
C3	0.059 (2)	0.067 (2)	0.041 (2)	−0.025 (2)	−0.008 (2)	0.007 (2)
C4	0.043 (2)	0.058 (2)	0.057 (2)	−0.0100 (19)	−0.004 (2)	0.018 (2)
C5	0.045 (2)	0.047 (2)	0.051 (2)	−0.0007 (18)	0.002 (2)	0.0018 (18)
C6	0.049 (2)	0.040 (2)	0.031 (2)	−0.0032 (17)	0.0062 (17)	−0.0026 (17)
C7	0.058 (2)	0.0411 (19)	0.039 (2)	0.0042 (17)	0.0026 (18)	−0.0027 (16)
C8	0.059 (2)	0.044 (2)	0.041 (2)	−0.0142 (18)	−0.0025 (18)	−0.0003 (17)
C9	0.050 (2)	0.045 (2)	0.045 (2)	−0.0105 (18)	−0.0053 (17)	−0.0001 (18)
C10	0.046 (2)	0.039 (2)	0.050 (2)	−0.0127 (16)	−0.0053 (18)	0.0023 (16)
C11	0.045 (2)	0.051 (2)	0.044 (2)	−0.0080 (17)	−0.0043 (16)	−0.0036 (17)
C12	0.058 (2)	0.0357 (19)	0.049 (2)	−0.0029 (16)	0.0037 (18)	−0.0009 (16)
C13	0.049 (2)	0.0341 (19)	0.031 (2)	−0.0020 (17)	0.0026 (17)	0.0038 (16)
C14	0.052 (2)	0.040 (2)	0.043 (2)	0.0028 (18)	0.011 (2)	−0.0008 (17)
C15	0.050 (2)	0.054 (2)	0.048 (2)	0.0009 (18)	−0.002 (2)	0.0006 (19)
C16	0.060 (2)	0.064 (2)	0.039 (2)	−0.007 (2)	−0.0029 (19)	0.0019 (19)
C17	0.077 (2)	0.053 (2)	0.039 (2)	0.000 (2)	0.011 (2)	−0.010 (2)
C18	0.054 (2)	0.0357 (19)	0.040 (2)	0.0063 (17)	0.0106 (19)	0.0029 (17)

### Geometric parameters (Å, °)

**Table:**

Cl1—C4	1.751 (3)	C15—C16	1.361 (4)
Cl2—C15	1.746 (3)	C16—C17	1.367 (5)
O1—C1	1.359 (3)	C17—C18	1.381 (4)
O2—C18	1.364 (3)	O1—H1	0.848
N1—C7	1.469 (3)	O2—H20	0.852
N1—C8	1.472 (3)	C2—H2	0.950
N1—C11	1.472 (3)	C3—H3	0.950
N2—C9	1.461 (3)	C5—H4	0.950
N2—C10	1.467 (3)	C7—H5	0.950
N2—C12	1.469 (3)	C7—H6	0.950
C1—C2	1.377 (4)	C8—H7	0.950
C1—C6	1.404 (4)	C8—H8	0.950
C2—C3	1.369 (4)	C9—H9	0.950
C3—C4	1.380 (5)	C9—H10	0.950
C4—C5	1.371 (5)	C10—H11	0.950
C5—C6	1.380 (4)	C10—H12	0.950
C6—C7	1.507 (4)	C11—H13	0.950
C8—C9	1.509 (4)	C11—H14	0.950
C10—C11	1.512 (3)	C12—H15	0.950
C12—C13	1.510 (4)	C12—H16	0.950
C13—C14	1.386 (4)	C14—H17	0.950
C13—C18	1.395 (4)	C16—H18	0.950
C14—C15	1.383 (4)	C17—H19	0.950
			
C7—N1—C8	110.6 (2)	C2—C3—H3	121.3
C7—N1—C11	111.9 (2)	C4—C3—H3	119.9
C8—N1—C11	108.6 (2)	C4—C5—H4	119.2
C9—N2—C10	109.8 (2)	C6—C5—H4	120.4
C9—N2—C12	111.2 (2)	N1—C7—H5	109.0
C10—N2—C12	112.1 (2)	N1—C7—H6	107.8
O1—C1—C2	118.5 (2)	C6—C7—H5	109.0
O1—C1—C6	121.9 (2)	C6—C7—H6	108.1
C2—C1—C6	119.5 (3)	H5—C7—H6	109.5
C1—C2—C3	121.3 (3)	N1—C8—H7	108.5
C2—C3—C4	118.8 (3)	N1—C8—H8	109.8
Cl1—C4—C3	120.0 (2)	C9—C8—H7	110.0
Cl1—C4—C5	118.9 (2)	C9—C8—H8	108.9
C3—C4—C5	121.1 (3)	H7—C8—H8	109.5
C4—C5—C6	120.3 (3)	N2—C9—H9	108.7
C1—C6—C5	118.8 (2)	N2—C9—H10	109.4
C1—C6—C7	120.9 (2)	C8—C9—H9	109.7
C5—C6—C7	120.2 (2)	C8—C9—H10	108.7
N1—C7—C6	113.4 (2)	H9—C9—H10	109.5
N1—C8—C9	110.2 (2)	N2—C10—H11	109.6
N2—C9—C8	110.9 (2)	N2—C10—H12	109.3
N2—C10—C11	110.0 (2)	C11—C10—H11	108.2
N1—C11—C10	110.5 (2)	C11—C10—H12	110.3
N2—C12—C13	112.4 (2)	H11—C10—H12	109.5
C12—C13—C14	120.3 (2)	N1—C11—H13	109.0
C12—C13—C18	121.3 (2)	N1—C11—H14	109.3
C14—C13—C18	118.3 (2)	C10—C11—H13	108.0
C13—C14—C15	120.3 (2)	C10—C11—H14	110.6
Cl2—C15—C14	119.1 (2)	H13—C11—H14	109.5
Cl2—C15—C16	119.8 (2)	N2—C12—H15	108.6
C14—C15—C16	121.0 (3)	N2—C12—H16	108.9
C15—C16—C17	119.3 (3)	C13—C12—H15	109.0
C16—C17—C18	121.1 (3)	C13—C12—H16	108.4
O2—C18—C13	120.9 (2)	H15—C12—H16	109.5
O2—C18—C17	119.1 (2)	C13—C14—H17	120.1
C13—C18—C17	119.9 (3)	C15—C14—H17	119.6
C1—O1—H1	107.2	C15—C16—H18	119.9
C18—O2—H20	107.0	C17—C16—H18	120.8
C1—C2—H2	119.1	C16—C17—H19	119.9
C3—C2—H2	119.5	C18—C17—H19	118.9
			
C7—N1—C8—C9	−178.0 (2)	C3—C4—C5—C6	1.4 (5)
C8—N1—C7—C6	167.4 (2)	C4—C5—C6—C1	−0.2 (4)
C7—N1—C11—C10	178.1 (2)	C4—C5—C6—C7	175.9 (2)
C11—N1—C7—C6	−71.4 (3)	C1—C6—C7—N1	−34.8 (3)
C8—N1—C11—C10	−59.5 (2)	C5—C6—C7—N1	149.3 (2)
C11—N1—C8—C9	58.7 (2)	N1—C8—C9—N2	−58.8 (3)
C9—N2—C10—C11	−57.8 (2)	N2—C10—C11—N1	59.6 (2)
C10—N2—C9—C8	57.8 (2)	N2—C12—C13—C14	−146.3 (2)
C9—N2—C12—C13	72.3 (3)	N2—C12—C13—C18	37.5 (3)
C12—N2—C9—C8	−177.5 (2)	C12—C13—C14—C15	−175.2 (2)
C10—N2—C12—C13	−164.3 (2)	C12—C13—C18—O2	−2.4 (4)
C12—N2—C10—C11	178.0 (2)	C12—C13—C18—C17	176.4 (2)
O1—C1—C2—C3	−178.2 (3)	C14—C13—C18—O2	−178.6 (2)
O1—C1—C6—C5	178.4 (2)	C14—C13—C18—C17	0.1 (3)
O1—C1—C6—C7	2.4 (4)	C18—C13—C14—C15	1.1 (4)
C2—C1—C6—C5	−1.2 (4)	C13—C14—C15—Cl2	176.9 (2)
C2—C1—C6—C7	−177.2 (2)	C13—C14—C15—C16	−1.6 (4)
C6—C1—C2—C3	1.4 (5)	Cl2—C15—C16—C17	−177.5 (2)
C1—C2—C3—C4	−0.3 (5)	C14—C15—C16—C17	1.0 (5)
C2—C3—C4—Cl1	178.6 (2)	C15—C16—C17—C18	0.2 (5)
C2—C3—C4—C5	−1.2 (5)	C16—C17—C18—O2	178.0 (3)
Cl1—C4—C5—C6	−178.4 (2)	C16—C17—C18—C13	−0.8 (5)

### Hydrogen-bond geometry (Å, °)

**Table:**

*D*—H···*A*	*D*—H	H···*A*	*D*···*A*	*D*—H···*A*
O1—H1···N1	0.85	1.88	2.649 (3)	150
O2—H20···N2	0.85	1.87	2.647 (3)	151
C7—H6···O2^i^	0.95	2.59	3.230 (3)	125
C12—H15···O1^ii^	0.95	2.57	3.300 (3)	134

Symmetry codes: (i) *x*, −*y*+1/2, *z*−1/2; (ii) −*x*+3/2, −*y*, *z*+1/2.
